# NOGO-A induction and localization during chick brain development indicate a role disparate from neurite outgrowth inhibition

**DOI:** 10.1186/1471-213X-7-32

**Published:** 2007-04-14

**Authors:** Shelley A Caltharp, Charmaine U Pira, Noboru Mishima, Erik N Youngdale, David S McNeill, Boleslaw H Liwnicz, Kerby C Oberg

**Affiliations:** 1 Department of Pathology and Human Anatomy, Division of Human Anatomy, Loma Linda University and Medical Center, Loma Linda, CA 92350, USA; 2 Department of Surgery, Division of Plastic and Reconstructive Surgery, Loma Linda University and Medical Center, Loma Linda, CA 92350, USA

## Abstract

**Background:**

Nogo-A, a myelin-associated protein, inhibits neurite outgrowth and abates regeneration in the adult vertebrate central nervous system (CNS) and may play a role in maintaining neural pathways once established. However, the presence of Nogo-A during early CNS development is counterintuitive and hints at an additional role for Nogo-A beyond neurite inhibition.

**Results:**

We isolated chicken *NOGO-A *and determined its sequence. A multiple alignment of the amino acid sequence across divergent species, identified five previously undescribed, Nogo-A specific conserved regions that may be relevant for development. *NOGO *gene transcripts (*NOGO-A*, *NOGO-B *and *NOGO-C*) were differentially expressed in the CNS during development and a second *NOGO-A *splice variant was identified. We further localized NOGO-A expression during key phases of CNS development by *in situ *hybridization. CNS-associated *NOGO-A *was induced coincident with neural plate formation and up-regulated by FGF in the transformation of non-neural ectoderm into neural precursors. NOGO-A expression was diffuse in the neuroectoderm during the early proliferative phase of development, and migration, but localized to large projection neurons of the optic tectum and tectal-associated nuclei during architectural differentiation, lamination and network establishment.

**Conclusion:**

These data suggest Nogo-A plays a functional role in the determination of neural identity and/or differentiation and also appears to play a later role in the networking of large projection neurons during neurite formation and synaptogenesis. These data indicate that Nogo-A is a multifunctional protein with additional roles during CNS development that are disparate from its later role of neurite outgrowth inhibition in the adult CNS.

## Background

Development of the central nervous system (CNS) is inherently complex. Neural plate induction is evident as thickening of epiblasts to form neuroectoderm. However, the precise interplay between the molecular pathways responsible for nervous system induction are less apparent. Early specification of central epiblasts to a neural fate is influenced by fibroblast growth factor (FGF) production while induction of non-neural epithelium in the periphery occurs through wingless-type (Wnt) and bone morphogenic protein (BMP) expression. FGF along with molecules that emanate from the organizer (Noggin, Follistatin and Chordin) later serves to limit BMP expression to the neural/epidermal border and thus maintain neural plate boundaries [1,2]. Maturation of the neural plate/neuroectoderm reenlists developmental pathways that are recycled to function as directors of proliferation, migration or establish repulsive/attractive environments within synaptogenesis. For example, after initial induction of the CNS, FGFs are subsequently re-utilized to maintain caudal progenitors by inhibiting differentiation [3], and later establish repulsive (FGF-8) versus attractive (FGF-4) environments to aid in directing cell migration [4]. Finally, FGFs are again used in CNS maturation by orchestrating neuronal networking through synaptogenesis [5].

**Figure 1:**
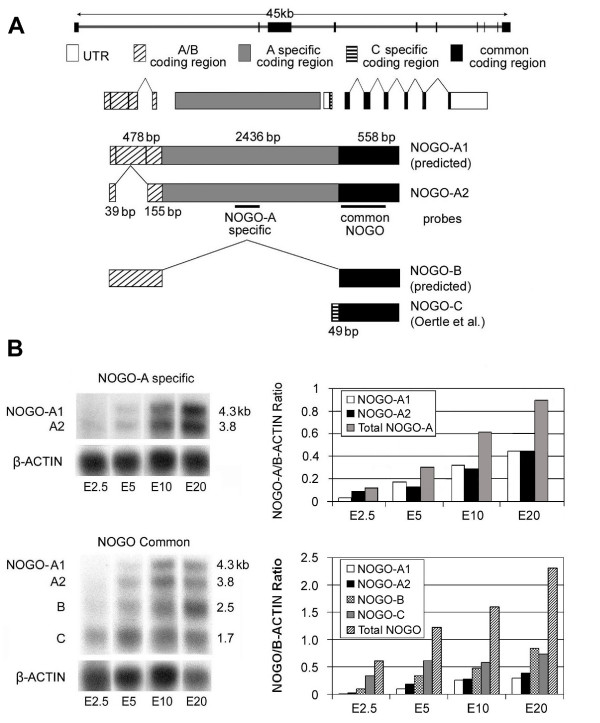
**Chicken *NOGO/RTN4 *gene structure and transcripts**. A) Gallus gallus *NOGO/RTN4 *gene localizes to chromosome 3 and produces four major transcription products. Isoforms are based on alternative splicing (*NOGO-A and B*) or promoter usage (*NOGO-C*). The predicted *NOGO-A1 *sequence is based on contig 96.95 (ensembl *Gallus gallus *genome) and is supported by our transcript size from northern analysis. *NOGO-A2 *results from alternative splicing in exon 1. *NOGO-B *is predicted based on transcript size from northern analysis and data from other species, while *NOGO-C *was previously isolated [20]. B) Northern analysis of *NOGO/RTN4*. *NOGO-A *specific probe identified two *NOGO-A *isoforms (A1, A2). Both isoforms increased with developmental age, the shorter form at slightly greater expression levels overall. A *NOGO *probe common to all isoforms (A, B, & C) confirmed progressive *NOGO-A *expression throughout development. Densitometry readings (graphs on the right) were normalized to *β-ACTIN*.

Once established, the CNS has limited regenerative capacity [6]. Inhibitory factors associated with myelin are released after CNS damage and block axonal outgrowth. Several potent myelin-associated inhibitors of neurite outgrowth have been isolated, including Nogo-A [7-9]. Antibody neutralization of Nogo-A allows axon growth into myelin [7] and identifies this factor as a critical endogenous inhibitor of CNS regeneration. *Nogo*/(*RTN4*) is a member of the endoplasmic reticulum-associated "reticulon" (RTN) family whose members have two hydrophobic, membrane spanning domains in the carboxyl-terminus. The *Nogo *gene gives rise to three main transcripts based on alternative splicing and variant promoter usage: *Nogo-A*, *Nogo-B *and *Nogo-C *(see figure 1). Between the two membrane spanning domains is a 66 amino acid extracellular loop that acts as a ligand to a specific Nogo66 receptor (NgR) [10]. However, only in Nogo-A, the largest transcript, has the receptor/ligand interaction been linked to neurite inhibition. Two other functional domains (an amino-terminal domain, NiRΔ2, and a Nogo-A specific domain, NiG-Δ20 [11]) have been identified that inhibit neurite outgrowth.

In adults, Nogo-A predominately localizes to oligodendrocytes [7,8] and can also be found at low levels in neurons of thalamic nuclei, cranial nerve nuclei and the Purkinje cell layer of the cerebellum [12-14]. In contrast, Nogo-A is widespread in fetal neurons [15-17]. Interestingly, the Nogo receptor (NgR) is absent in the embryonic and fetal brain [18]. Thus, the presence of Nogo-A during CNS development when regeneration is possible [19] and NgR is absent, hints at an additional role for Nogo-A.

In this report we describe the induction and temporospatial expression of *NOGO-A *from the earliest stages of CNS development with the induction of neuroectoderm. We also describe a progressive increase in *NOGO-A *expression with chick brain development. We further localized and correlated this progressive increase in *NOGO-A *expression to specific cell types and key events of CNS development within the highly organized chick optic tectum. Collectively, our results suggest a fundamental role for Nogo-A in CNS development and maturation that is unrelated to its later role as an inhibitor of axonal regeneration.

## Results

### Isolation and northern analysis of chicken NOGO-A

We isolated and cloned the chicken *NOGO-A *coding and 3' untranslated region. The *NOGO-A *sequence aligned to chromosome 3 of the chicken genome with 10 exons spanning 45 kb (Fig 1A). Sequence comparison with the chicken genome revealed the *NOGO-A *sequence to be lacking 248 bp within the first exon. Given that *NOGO-A *and *-B *isoforms originate from alternative splicing, we deduced our isolated sequence to be a shorter splice variant, classified as *NOGO-A2*. Northern analysis with a *NOGO-A *specific probe (see probes in Fig 1A) confirmed the secondary splice product. The larger *NOGO-A1 *and *NOGO-B *isoforms were predicted based on the chicken genome and northern hybridization. The chicken *NOGO-C *sequence was described previously [20].

To determine the expression profile of *NOGO-A *during CNS development, we used the *NOGO-A *specific probe for northern analysis. Hybridization with poly-A RNA isolated from chick brains of HH15 (E2.5), HH25 (E5), HH36 (E10) and HH45 (E20, just prior to hatching) revealed a 4.3 kb band (*NOGO-A1*), consistent with *NOGO-A *transcripts in other species [7-9], and a shorter 3.8 kb band (*NOGO-A2*) (Fig 1B). At the earliest stage (HH15) the longer *NOGO-A1 *transcript was nearly absent. Overall, combined *NOGO-A *expression increased with development reaching a maximum just prior to hatching (E20).

To ascertain whether *NOGO *isoforms were differentially expressed during development, we designed a probe to the 3' end of *NOGO *which was common to the *NOGO-A*, *NOGO-B *and *NOGO-C *isoforms and performed northern blot analysis of the same embryonic stages as above. In agreement with our previous data, 4.3 kb and 3.8 kb transcripts were found corresponding to *NOGO-A1 *and *A2*. In addition, 2.3 kb and 1.7 kb bands corresponding to the *NOGO-B *and *NOGO-C *isoforms were also identified (Fig 1C). Collectively, *NOGO *transcription increased with development. The *NOGO-A *profile demonstrated by the common probe was the same as that of the *NOGO-A *specific probe. The progressive changes in *NOGO-B *expression were similar to *NOGO-A *at early stages, but showed a marked increase at E20. In contrast, *NOGO-C*, which localizes to neurons and mesenchymal tissues, exhibited strong somewhat uniform expression throughout development. Northern analyses were quantified by densitometry of the autoradiographs and normalized to *β-ACTIN *levels (Fig 1B &1C-graphs).

### Identification of Evolutionarily Conserved Regions

**Figure 2:**
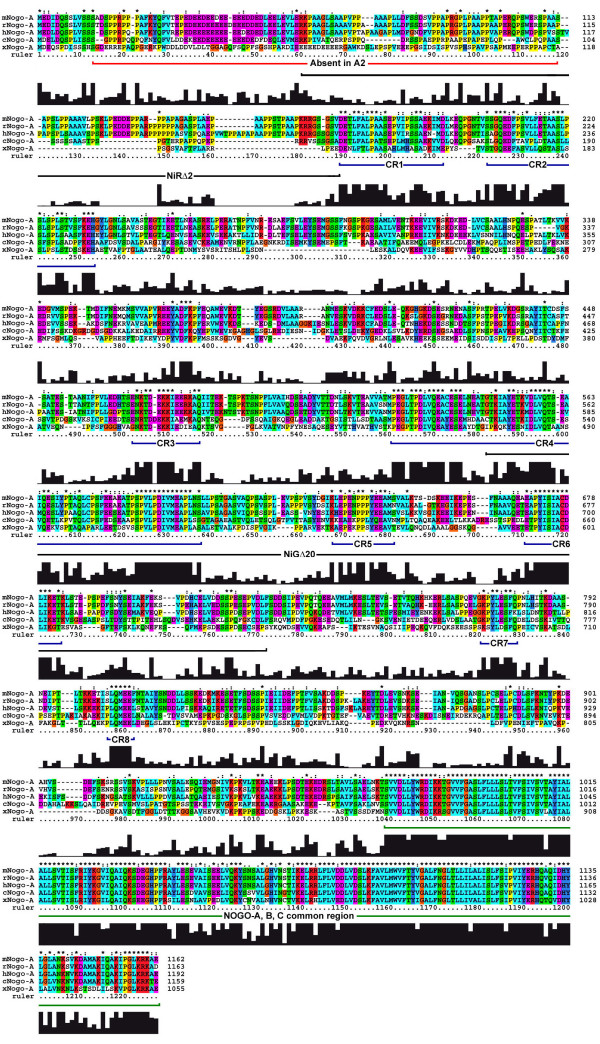
**Multiple sequence alignment of Nogo-A/Rtn4-A protein**. The multiple sequence alignment compares Nogo-A protein from mouse, rat, human, chicken and xenopus (Rtn4.2-A1). Putative Nogo-A specific functional domains based on conserved regions (CR1-8) having ≥ 70% for 7 or more amino acids with a sequence identity/strong group conservation. Inhibitory domains NiRΔ2 and NiGΔ20 were previously identified [11]. Amino acid color code: Red-KR, Orange-G, Yellow-P, Green-NQST, Cyan-ACFILMVW, Blue-HY, Magenta-DE. "*" denotes conserved residue; ":", conservation of a 'strong' group; ".", conservation of a 'weaker' group as defined by ClustalX (see methods) [Genbank: AY494005 and AY843529].

Multiple alignment of the chicken NOGO-A shared 49%, 47%, 46% and 32% identity with human, mouse rat and xenopus (Rtn4.2-A1), respectively. The C-terminal 189aa, i.e., the common NOGO/Reticulon region, exhibited significant conservation (>75%) consistent with what has been previously reported [20]. In addition, eight conserved regions (CR) were found within the NOGO-A specific coding sequence, suggesting potential functional domains (Fig 2). Conserved regions had ≥ 70% for 7 or more amino acids with a sequence identity/strong group conservation. Strong group refers to amino acids with similar chemical structure and function. Interestingly, there was no significant conservation found within the first two coding regions shared by NOGO-A and -B isoforms, although this region corresponds to a previously identified inhibitory domain in rat, NiRΔ2 [11]. Rather, the first conserved region (CR1) initiated at the first amino acid residue of the NOGO-A specific coding region. Additionally, CR4-6 correspond to a potent inhibitory domain, NiGΔ20 [11].

### Expression During Neurulation

**Figure 3:**
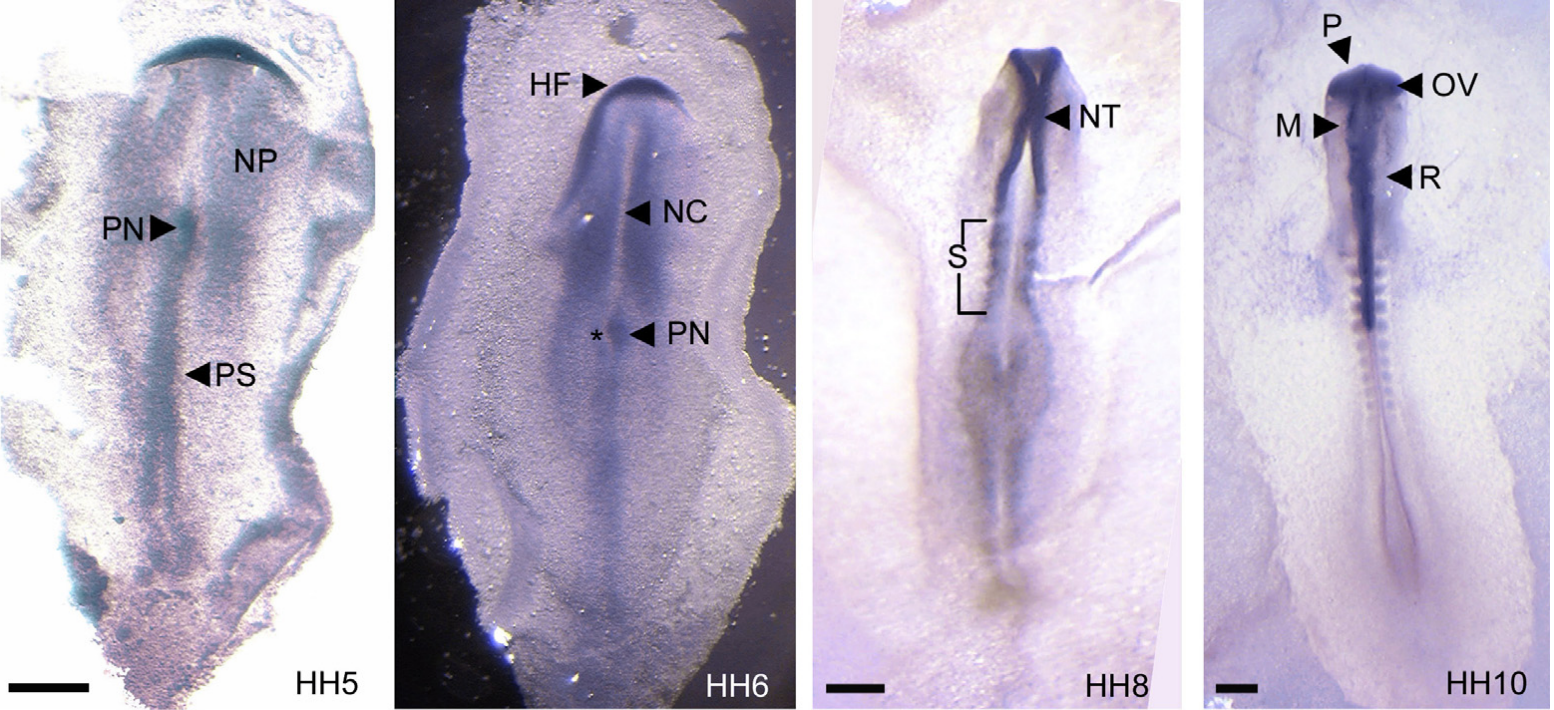
**Whole mount in situ hybridization (WISH) of early chick embryos**. *NOGO-A *expression localized to the neural plate (HH5), and was excluded from the notochord. Early expression was also evident within the primitive node and streak. Interestingly, *NOGO-A *is asymmetrically expressed within the primitive node (*). *NOGO-A *expression demarcated the head fold (HH6), neural tube (HH8) and brain vesicles (HH10). Expression was also present within developing somites (HH8 and HH10). HF, head fold; M, mesencephalon; NC, notochord; NP, neural plate; NT, neural tube; OV, optic vesicle; P, prosencephalon; PN, primitive node; PS, primitive streak; R, rhombencephalon; S, somite. Scale bars 250 μm.

The quantitative limitations of northern analysis precluded our ability to detect *NOGO-A *expression at the earliest stages of chick CNS development. Thus, we used whole mount *in situ* hybridization (WISH) to detect and localize low levels of transcript expression to developing structures and cell layers. *NOGO-A *gene expression was detected as early as HH3 within the primitive streak and node (as shown in HH5, Fig 3) and remained within these organizing structures until their regression. *NOGO-A *subsequently localized to the forming CNS during neural plate induction (HH5 Fig 3). Expression was lacking in the notochord and overlying neuroectoderm of the presumptive neural groove (HH6). Furthermore, expression was asymmetric within the primitive node, being restricted to the right side. *NOGO-A *expression increased during neural tube formation (HH8, Fig 3) and attained the full length of the body axis within the primitive spinal chord by HH13. Also of note was the focal presence of *NOGO-A *associated with forming somites (starting at HH7-8, Fig 3).

### Expression during Tectal Development

Spatiotemporal expression of *NOGO-A *during pivotal stages of tectal development was next characterized. The expression was then correlated with stage-specific functional markers by IHC.

#### During Cellular Proliferation

**Figure 4:**
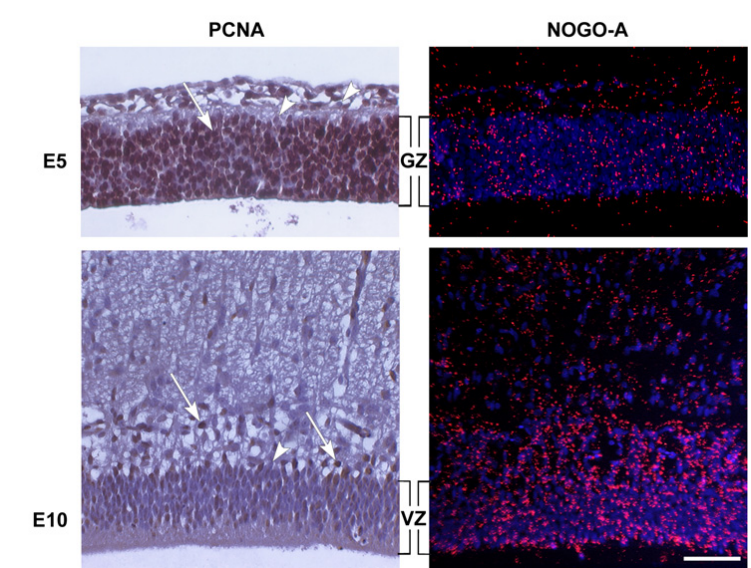
**Comparison of *NOGO-A *expression to proliferative activity**. HH25 (E5) chick optic tectum showing Proliferating Cell Nuclear Antigen (PCNA) immunoreactivity. Section in situ hybridization shows diffuse *NOGO-A *expression (red) at low levels throughout the proliferative, periventricular neuroepithelium (generative zone, GZ). At HH35 (E10), there is low PCNA staining within the periventricular neuroepithelium, now termed ventricular zone (VZ), while *NOGO-A *expression has increased (arrowheads highlight PCNA negative nuclei, while arrows point out PCNA positivity). Scale bar 200 μm.

Neuroepithelial proliferation peaks at HH26 (E5); by HH36 (E10) proliferation decreases to minimal levels [21]. To determine whether NOGO-A expression is related to proliferative activity, we compared expression at HH26 and HH36. At HH26, the primitive optic tectum is composed of a psuedostratified layer of neural ectoderm, i.e. the generative zone (GZ). *NOGO-A *expression was weak and diffuse throughout the GZ. In contrast, proliferative cell nuclear antigen (PCNA) staining was intense within most nuclei (Fig 4, E5). At HH36, *NOGO-A* expression increased within the residual neuroepithelium, now termed ventricular zone (VZ), whereas PCNA staining is nearly absent in the post-proliferative VZ (Fig 4, E10).

#### During Cell Migration

**Figure 5:**
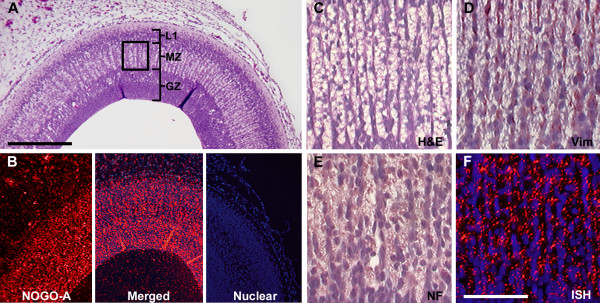
***NOGO-A *expression during cell migration**. A) H&E staining of optic tectum at HH30(E7) showing the generative zone (GZ), the migrating zone (MZ) and the first neuronal lamina (L1). B) Section in situ hybridization (sISH) of NOGO-A in an adjacent section demonstrating a corresponding radial pattern of expression in the region of migration. High magnification of the MZ (from boxed region in "A") shows the elongated processes of radial glia and associated migrating neurons. C) H&E for morphologic comparison; D) vimentin (Vim) immunohistochemistry (IHC) decorating the intermediate filaments of the radial glia; E) IHC of Neurofilament (NF) targeting migrating neurons; and F) sISH showing NOGO-A expression (red) more closely resembling NF staining. Scale bar 200 μm in A, B; 50 μm in C, D, E, F.

The eventual stratified organization of the tectum mandates reorganization of the proliferating neuroectoderm through migration and sequential lamination. To evaluate the expression of *NOGO-A *during early lamination, we examined the optic tectum at HH30 (E7). At this stage there is an undifferentiated layer of proliferating neuroepithelium (the generative zone, GZ), an expanding migratory zone (MZ), and the first neuronal lamina (L1) (Fig. 5A). *NOGO-A *expression could be distinguished within all layers of the E7 tectum (Fig 5B). To differentiate the expression of *NOGO-A *between neurons and the supporting cells of the MZ, specifically radial glia, we performed IHC for neurofilament-M and vimentin, respectively. Magnified views of the MZ (Fig 5C–F) show *NOGO-A *expression more closely resembled the neurofilament staining of the neuronal nuclei and their delicate processes (Fig. 5E and 5F). However, the intensity of *NOGO-A *expression was similar in all three zones and did not appear to be limited to actively migrating neurons within the MZ (Fig 5B).

#### During Network Establishment

**Figure 6:**
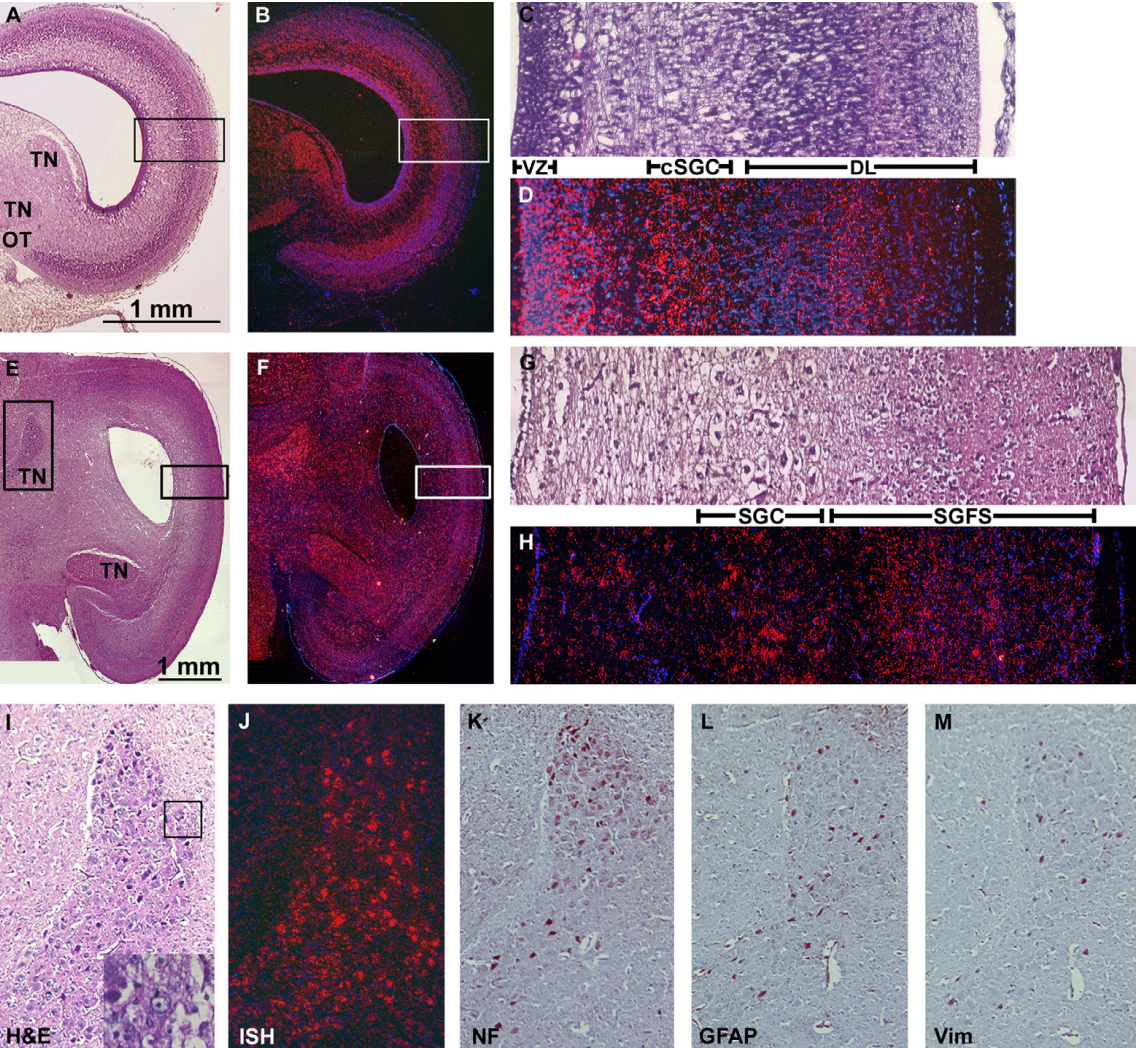
***NOGO-A *Expression During Network Establishment**. A) H&E and B) *NOGO-A *in situ hybridization (ISH) of HH36 (E10) optic tectum, prior to synaptogenesis. Transcript expression is intensified in cells of the tectal associated nuclei (TN) and cortical neuronal lamina. Note the lack of expression in the fibers of the optic tract (OT). C&D) Magnified view of tectal lamination (horizontal boxes) from A&B reveal the presumptive projection layer of the tectum, the compartment *stratum griseum central *(SGC), as the cortical neuronal lamina strongly expressing *NOGO-A*. Ventricular Zone-VZ, developing lamina-DL. E) H&E and F) *NOGO-A *in situ hybridization at E20 (after significant synaptogenesis) shows punctate expression localized to the large neuronal cell bodies of the tectal associated nuclei, the SGC, and to deep cellular layers of the main retinal afferent layer, the *stratum griseum et fibrosum superficiale *(SGFS). G&H) Magnified view of tectal lamination (horizontal boxes) from C&D. I-M) Serial sections localizing *NOGO-A *expression to projection neurons within a tectal associated nucleus (vertical box in C). I) H&E showing the large cell bodies of the projection neurons (inset showing large cytoplasm-rich neuron with nuclear clearing and prominent nucleolus), J) punctate *NOGO-A *expression, K) immunohistochemistry (IHC) of neurofilament (NF), highlighting neuronal cell bodies, L) IHC of glial fiber associated protein (GFAP) demonstrating glial supporting cells, and M) IHC for vimentin (Vim) demarcating a few immature glial cells.

To evaluate the expression of *NOGO-A *during network establishment, i.e. neuronal maturation, fiber growth, and synaptogenesis, we compared expression patterns of pre-synaptogenic (E10) and post-synaptogenic (E20, just prior to hatching) stages. At E10, prominent *NOGO-A *expression was present at the periphery of the VZ and radiated towards the cellular cortex (Fig 6A, B-horizontal box). Magnified view of *NOGO-A *in the tectal cortex showed heightened expression within the compartment *stratum griseum centrale *(cSGC), the presumptive layer of tectal projection neurons and the only well defined compartment at this stage (Fig 6C,D). *NOGO-A *is not a marker of cellularity as the SGC is cell poor relative to adjacent developing lamina (DL). Expression was also increased throughout cells of the forming tectal-associated nuclei (TN, Fig 6A), in contrast to the nearby fibers of the unmyelinated optic tract (OT).

By E20, the tectum has formed the basic architecture with all lamina represented. *NOGO-A *expression does not appear to have increased with expansion and maturation of the tectum (Fig 6E,F). Rather, a redistribution of expression to larger neurons has occurred throughout the tectum and tectal-associated nuclei. At higher magnification, *NOGO-A *expression continued to be accentuated within somata of larger neurons, as again exemplified by multipolar neurons of the SGC (Fig 6G,H). Increased expression was also present within some of the larger neurons of the now formed multi-laminated *stratum griseum et fibrosum superficiale *(SGFS), the major retinal afferent layer of the tectum.

Immunohistochemistry for neurofilament (NF) in a tectal associated nuclei, the *nucleus spiriformis lateralis*, co-localized *NOGO-A *with large neurons typical of tectal associated nuclei while stains for astrocytes (GFAP) and glial progenitor cells (vimentin) showed less overlap with *NOGO-A *(Fig 6I–M). Although *NOGO-A *expression correlated best with these large neurons, it should be noted that *NOGO-A *could also be seen at low levels in cell bodies that were neither NF, vimentin nor GFAP positive, which likely represent differentiating oligodendrocytes.

#### During myelination of axon tracts

**Figure 7:**
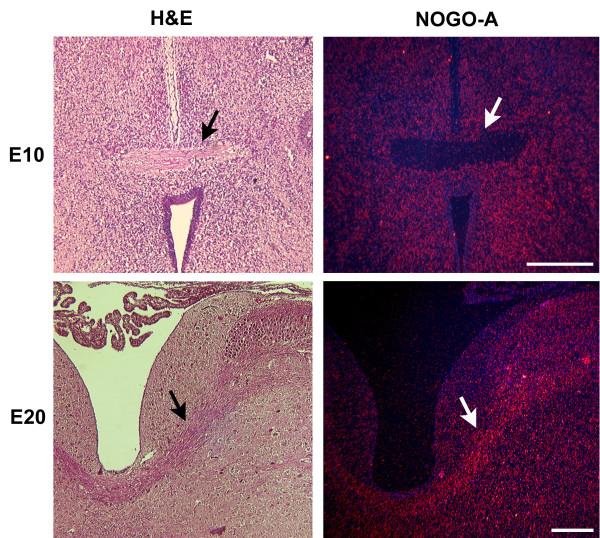
**Oligodendrocyte-related *NOGO-A *expression within axon tracts**. H&E of an axon tract (arrow) within the E10 chick brain preceding oligodendrocyte related myelination. Section in situ hybridization (sISH) of the fiber tract shows *NOGO-A *expression to be absent within the tract but present in surrounding neurons. H&E of a myelinated axon tract (arrow) at E20. ISH shows oligodendrocyte related *NOGO-A *expression within the fiber tract. Scale bars 250 μm.

The transition between network establishment and maintenance is coupled to myelination of established axonal tracts. Oligodendrocyte differentiation within the chick tectum occurs between E12–E17 [22]. To determine the correlation of increased *NOGO-A *expression within axon tracts and oligodendrocyte related myelination, we again looked at the E10 and E20 chick brain, stages which precede and follow oligodendrocyte differentiation. Axonal tracts of the E10 chick brain are noticeably devoid of *NOGO-A *expression in comparison to the surrounding *NOGO-A *positive neurons (Fig 7). In contrast, the E20 brain reveals significant *NOGO-A *expression within the axonal tracts relative to the surrounding tissue.

### *NOGO-A* is Up-regulated by FGF4

**Figure 8:**
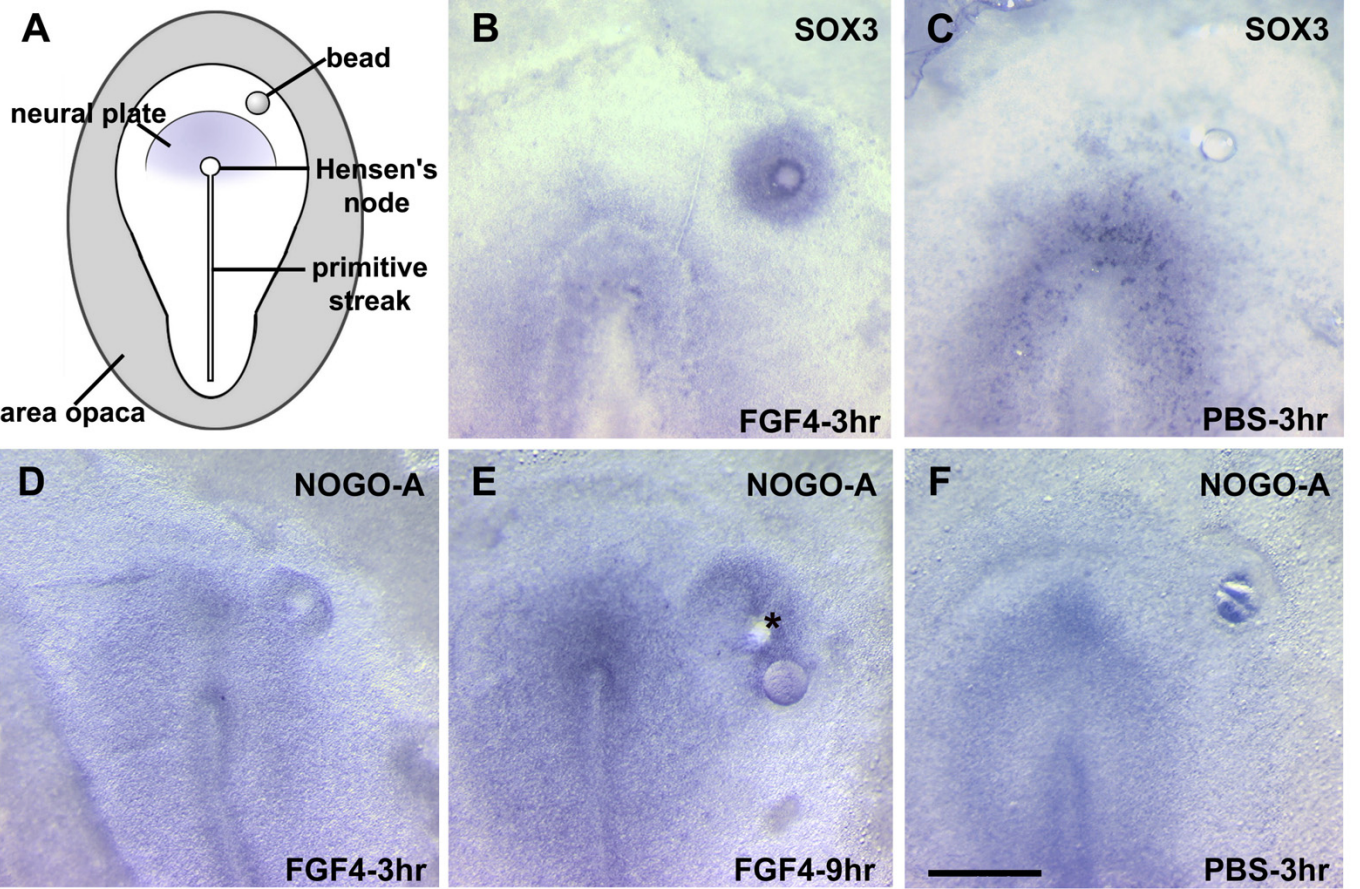
**Regulation of *NOGO-A *by FGF in the early chick embryo**. A) Schematic of an early primitive streak stage chick embryo (HH4) illustrating the location of bead placement (anterior-right) within the area pellucida between the neural plate and the area opaca (outer gray). B) FGF4 upregulation of a pre-neural marker, *SOX3*, 3 hours after application. C, F) PBS-soaked beads failed to induce the pre-neural marker or *NOGO-A*. D, E) *NOGO-A *induction following FGF4 application was upregulated after 3 hours and expression was maintained around the bead following 9 hours of incubation. Scale bar 250 μm. "*" initial position of bead in E) prior to processing.

Because *NOGO-A *expression coincides with the formation of the neural plate, we evaluated whether *NOGO-A *could be induced in cranial non-neural epiblasts in response to ectopic application of FGF (Fig 8A), an early initiator of neural programming. A number of FGFs are expressed during neural induction (FGF2, FGF3, FGF4, FGF8) and might play a role in the initiation of the neural program. FGF4 is often used in overexpression assays because it causes neural induction at a significantly lower concentration than other FGFs. This is believed to be due to the fact that FGF4 binds to a number of FGF receptors and is likely to reproduce the activity of other FGFs [23,24]. We found that *NOGO-A *expression was up-regulated around beads soaked in FGF4 (50 μg/ml) three hours after application in HH 3^+^/4 chick embryos (6 out of 6 embryos). This rapid FGF induced up-regulation of *NOGO-A *was equivalent to *SOX3*, an early pre-neural marker (Fig 8B). *NOGO-A *expression persisted around the bead for at least 9 hours (last time point examined, 5 out of 6 embryos). PBS control beads did not up-regulate either *SOX3 *or *NOGO-A *(Fig 8C,F).

## Discussion

A role for Nogo-A as an inhibitor of CNS regeneration is well known, where expression is restricted to the axon ensheathing oligodendrocytes [7-10]. Nogo-A found within the plasma membrane of oligodendrocytes inhibits neurite outgrowth by promoting growth cone collapse. It was unexpected, therefore, when we and others found this inhibitory protein expressed during CNS development, and in particular, within developing neurons [15-17,19,25,26]. Our study profiles the expression of *NOGO-A *as it relates to key phases of CNS development: induction, proliferation, migration/differentiation, and network establishment. While the embryonic expression of *NOGO-A *has been reported [15,25,26], most fetal studies have focused on its role as an inhibitor of CNS regeneration [17,18,13,27]. Our results imply that during development Nogo-A has a function that is different from its "adult" role of inhibiting CNS regeneration.

### Conservation of the Nogo-A Sequence

When aligned with human, rat, mouse and xenopus amino acid sequences, the chicken NOGO-A sequence has a high degree of identity (>75%) in the carboxyl-terminal, reticulon-specific portion that is present in all three major isoforms (A, B and C). Multiple alignment of widely different species also revealed eight conserved regions (CR1-8) within the Nogo-A specific coding sequence (Fig 2). This conservation implies preservation of domains that are functionally significant. Previous functional domains have been identified in regions common to all Nogo isoforms [Nogo66 in the C-terminus, NiRΔ2 (rat-aa 59–172) in the shared Nogo-A/B regions], and in the Nogo-A specific region [NiGΔ20 (rat-aa 544–725)], but have been linked to inhibitory roles in neurite outgrowth and cell spreading [11]. The NiGΔ20 inhibitory domain overlaps three highly conserved regions identified in our analysis (CR4-6) suggesting a conserved function across taxa. Interestingly, NiRΔ2 does not correspond to one of the highly conserved regions and previous reports have demonstrated poor preservation of this region in amphibians [28]. Collectively, these data may indicate a mammalian specific role for NiRΔ2. Several highly conserved regions remain in this long protein without known functional roles. It is likely that one or more of these regions are important for Nogo-A's participation in development, however, further studies are needed to validate this prospect.

### Differential Regulation of *NOGO* during Development

Expression of the *NOGO-C *isoform was broad and persistent in the developing brain by northern analysis and was in accord with adult expression profiles, where *NOGO-C *can be found in neurons as well as skeletal muscle [7]. The larger (*NOGO-A *and -*B*) isoforms showed progressive expression with development (Fig 1C). Interestingly, we identified two *NOGO-A *transcripts in the chick, which confirms an earlier report identifying two NOGO-A bands by immunoblot [19]. Sequence analysis against the published chicken genome verified the two bands as alternative splice products, with the smaller band resulting from a secondary splice site in exon 1, similar to what is observed in Xenopus [29]. It is unclear what functional advantage these two splice variants offer, however, there appears to be differential stage-specific expression by northern analysis which suggests stage-specific roles.

### A Role for Nogo-A in CNS induction

The onset of neurulation is defined by formation of the neural plate. *NOGO-A *is induced in the neural plate during its formation and persists in structures derived from neuroectoderm. Furthermore, our data demonstrate that transformation of non-neural ectoderm into presumptive neuroectoderm by ectopic FGF is characterized by rapid induction of SOX3 (a "pre-neural" marker) and *NOGO-A *(Fig 8B,E). Recent microarray analysis supports this finding with the identification of *NOGO-A *in human and porcine neural precursor cells [30]. These findings suggest that Nogo-A participates in specifying neuronal identity and/or differentiation.

However, non-neuronal *NOGO-A *expression was also present in the primitive streak and node, structures which precede neural plate formation (HH5, Fig 3). *NOGO-A *expression persists within these structures until their regression. This differential induction indicates tissue specific and temporal-spatial regulation of *NOGO-A*. Interestingly, there was asymmetrical right-sided *NOGO-A *expression within the primitive node (Hensen's node) and scant expression in the notochord and presumptive neural groove immediately overlying the notochord. This unique expression pattern is complimentary to the expression of Sonic Hedgehog (SHH), a morphogen critical to patterning of the developing neural tube (See additional file 1). *NOGO-A *also exhibits focal expression associated with somite formation at what may be the sites of future dorsal root ganglia and spinal nerves. We and others have shown that dorsal root ganglia have robust *NOGO-A *expression (See additional file 2) [15,13].

The cells of the neural plate, primitive streak/node and somites all demonstrate rapid growth and patterned structural transformations. It is possible that Nogo-A may have neural-specific roles and additional broader roles related to morphogenesis. Changes in cell shape and migration are orchestrated by cytoskeletal reorganization. Nogo-A signaling has been linked to the downstream activation of the small GTPase, RhoA [31]. In development, the Rho family, through Rho kinases, regulates cytoskeletal reorganization associated with changes in cell shape such as the formation of axons and dendrites [32-34]. Moreover, the published expression of *Rho Kinase α *[35] overlaps the expression of *NOGO-A *shown here. Inhibition of Rho kinases results in disrupted formation of the brain/neural tube, reduced caudal extension of the primitive streak and loss of left-right asymmetry. The asymmetric expression of *NOGO-A *within the primitive node, and its expression within the primitive streak may indicate an additional role for this protein in organogenesis and tissue identity.

### Additional roles in later CNS development

Many molecules critical to CNS development have multiple functions. Molecules such as FGFs, SHH, BMPS and Wnts, once thought to play isolated roles in development, have now also been linked with several steps in neurulation and CNS development including formation of the neuronal circuitry through axon guidance and synaptogenesis [36]. The expression of *NOGO-A *at key phases of CNS development from neural induction to definitive network establishment suggests that this factor may also have multiple functions.

To examine potential roles of Nogo-A during specific phases of CNS development we utilized the chick optic tectum. This highly structured region serves as an ideal model for understanding formation of the vertebrate brain, attaining a high level of complexity while completing most of its maturation prior to hatching. *NOGO-A *was present in the tectum at all stages of embryonic development observed, nevertheless, clear patterns emerged when looking at embryonic stages correlating to the specific phases of CNS development.

### Nogo-A during Proliferation and Migration

Proliferation is a critical part of early development of the expanding neural tube. We saw no correlation between *NOGO-A *expression and Proliferative Nuclear Cell Antigen (PCNA) positivity when comparing neuroepithelium during peak proliferative and relative quiescent stages (E5 vs E10, respectively) [37]. Thus, it is unlikely that Nogo-A has a role in proliferation.

From E6 onward a migratory zone of post-mitotic neurons can be visualized in the tectum [38]. *NOGO-A *expression in the E7 tectum was homogenously expressed across the pre-migratory generative zone, the expanding migratory layer, and the post-migratory, first neuronal lamina. Immunostaining for neuronal and glial cells linked *NOGO-A *expression to the soma of neurons, however, *NOGO-A *expression was not limited to the neuronal population actively undergoing migration. Thus, our data does not support a role for Nogo-A limited to directing the migration of neurons. Rather, the continued expression of *NOGO-A *within all populations of developing neurons is further support of a role marking early neural identity and may be related to the state of maturity/differentiation. This concept is in agreement with previous studies that reported a high neuronal *NOGO-A *mRNA expression during differentiation of the spinal cord [15], and a down regulation of *NOGO-A *mRNA following migration and terminal differentiation of rat olfactory neurons [25] and cerebellar granule cells [17].

### Nogo-A during Neuronal Differentiation

Neuronal differentiation is also characterized by the outgrowth of neurites and the formation of synapses. Neuritogenesis and synaptogenesis coupled with later refinement of connections by cell death and neurite pruning comprise the process of "network establishment." The *Stratum Griseum Centrale *(SGC) of the chick optic tectum is sparsely populated by large, multi-polar principal efferent neurons with extensively branched dendrites and robust projecting axons [39]. By E10, extension of axons and dendrites is abundant in the SGC and intense expression of *NOGO-A *can be seen within these large neurons. Heightened *NOGO-A *expression can also be seen in nearby tectal associated nuclei, which are conspicuous by their large, projecting neurons.

By E14, the first synaptic junctions of the tectum are observed. However, their numbers rise drastically between E18 and the first hours after hatching with more intense retinal input [40]. Loss of retinal input to the tectum can be disturbed by embryonic enucleation of the chick eye optic anlagen. Deafferentation causes neuronal degeneration of the SGC through loss of synaptic input and prevention of forming synaptic connections [41]. Correspondingly, there is an overall decrease in *NOGO-A *expression within the SGC and the tectal associated nuclei suggesting *NOGO-A *expression is dependent upon synaptic activity (Caltharp, unpublished data).

Increased Nogo-A immunoreactivity has been demonstrated at the onset of axon growth in developing rat olfactory neurons [25] and also within sprouting dendrites of cerebellar Purkinje cells [17]. Interestingly, over expression of Nogo-A within COS cells leads to the formation of long processes that resemble neurites [42]. As mentioned earlier, Nogo-A can regulate RhoA dependent cytoskeletal reorganization which is intensely active during neuronal differentiation and neuritogenesis. Collectively, these data combined with our findings provide strong support for a Nogo-A specific role in neuronal differentiation and neuritogenesis.

### Nogo-A during the Transition to Adulthood

At E10, *NOGO-A *expression within the tectum is strictly neuronal. Oligodendrocyte related myelination in the chick tectum takes place between E12–E17 [22] and accordingly, unmyelinated fiber tracts within the tectum are distinctive for an absence of *NOGO-A*. By E20, these same fiber tracts have begun to acquire myelination by oligodendrocytes and are now positive for *NOGO-A *expression. Studies marking glial differentiation found Nogo-A to be expressed at all stages of oligodendrocyte development [18,13] and our data found *NOGO-A *expression within cells of tectal associated nuclei that were neither positive for neuron, astrocyte or glial progenitor specific stains. This later *NOGO-A *expression within smaller cells of the tectum is a likely sign of increased oligodendrocyte formation and may reflect the beginning transition of expression from neuronal differentiation and network establishment to myelin derived maintenance of the circuitry.

## Conclusion

In this study, we have demonstrated *NOGO-A *induction to be coincident with the earliest indication of the CNS, neural plate formation. Furthermore, *NOGO-A *expression persists in neurons throughout development. *NOGO-A *is also upregulated by FGF which is known to transform ectoderm into neuroectoderm. Collectively, these data support a functional role for Nogo-A linked to neural identity.

Accentuated *NOGO-A *expression is later observed within large projection neurons, as exemplified by neurons of the *stratum griseum centrale *in the optic tectum. Projection neurons are characterized by extensive dendritic branching and broad prominent axons. *NOGO-A *expression peaks during the formative stage of this lamina (E10) and may indicate an additional role for Nogo-A in neuritogenesis and axon branching.

The unique portion of the chicken NOGO-A isoform is 800 amino acids in length and contains 8 conserved regions (CR) across five species (spanning from xenopus to human). Three of these conserved regions (CR4-6) correspond to a previously identified inhibitory domain (NiG20). This leaves 5 conserved regions that may represent important Nogo-A specific functional domains relevant to development. Although conservation suggests functional importance, further studies are needed to validate this premise.

## Methods

### Probe isolation and Bioinformatics

Within a region specific for Nogo-A, amino acid sequences conserved between human and rat were identified and used to generate a 5' degenerate primer (5'-GCCTGAAGGYCTGACKCC-3') and a 3'degenerate primer (5'-GGTGCTTCCATAACAATRTCAGGC-3'). These primers were used to isolate a 219 bp fragment of chicken *NOGO-A *[Genbank: AY494005] from embryonic chick brain cDNA (E10). This fragment was cloned into plasmid vector pCRII-Topo (Invitrogen) with the capacity for forward and reverse transcription. 3' extension of the initial isolated fragment was performed using the designed 5'p gene specific primer and an Oligo-dT primer as provided in the GeneRacer Kit (Invitrogen) for amplification, cloning and sequencing of the cDNA. A probe for the common region of the *NOGO *gene was based on sequencing results of 3' *NOGO-A *using the common 5' primer (5'-gttgttgacctcctttactgg-3') and common 3' primer (5'-ccagtatcagtaatgtcagacc-3'). The resulting fragment was cloned as described above.

For complete 5' sequencing of *NOGO-A*, we constructed primers based on the chicken genome (Gallus_gallus 1.0[43]) to regions that appeared to match start sites of the gene within other species. Triple Master (Eppendorf) GC rich PCR protocol with 4% DMSO was used for sequence isolation [Genbank: AY843529]. Protein multiple alignment was performed using Clustal X (version 1.83) as described [44]. Regions of significant conservation were determined by receiving a score of 70% sequence similarity based on amino acid identity (as indicated by asterisk) and/or conservation of one of the 'strong' groups (as indicated by colon), see [44]. The alignment was run on a downloaded program [45].

### Chicken embryos

White Leghorn fertilized eggs were obtained from Hyline International (Lakeview, CA). Eggs were incubated at 39°C in a humidified chamber, and embryonic age was determined according to Hamburger and Hamilton's (HH) staging system [46]. Chicks representing stages 3–11,15, 17, 25, 30, 33, 36, and 45 were isolated for study.

### Whole mount *In Situ* Hybridization (wISH)

Chicks at stages 3 to 11 (14 hr to 45 hr incubation) were harvested with a filter paper ring support, rinsed in cold PBS and fixed in MEMFA pH 9 (0.1 M MOPS pH7.4; 2 mM EGTA; 1 mM MgSO_4_; 3.7% formaldehyde) overnight at 4°C. Embryos were then briefly rinsed in PBS, transferred to ice cold 90% methanol and stored at -20°C until processed. Anti-sense riboprobe from the 219 bp *NOGO-A *fragment was generated from linearized plasmid using digoxigenin-tagged rUTP (Roche) in the substrate mix following standard whole mount *in situ* protocol [47]. Plasmids for *SOX2* and *SOX3* probes were kind gifts from Drs. Domingos Henrique, R. Lovell-Badge and P. Scotting. Subsequent steps were carried out either manually for the earliest stages (3–11) or with the InsituPro automated whole mount ISH (Intavis AG) for stages 15 and 17. Embryos were rehydrated and treated with proteinase K (10 μg/ml) for 5 min. Probe was applied overnight (~12 hr) at 58°C and post hybridization washes were carried out at 63°C. After pre-blocking embryos with 2% blocking reagent (Roche), a high-affinity anti-digoxigenin antibody conjugated to alkaline phosphatase (Roche) was applied. Colorization reaction with BCIP-NBT in 12.5% PVA was performed in the dark and checked periodically under a dissecting microscope until reaction was deemed complete (approximately 2–3 hr).

### Section *In Situ* Hybridization (sISH)

Whole embryos of chicks at stages 15 and 25 (E 2.5 and 4.5, respectively) and dissected brains from chicks at stages 30, 33, 35 and 45 (E 7,8, 10 and 20, respectively) were harvested, fixed in 4% paraformaldehyde (PFA) for 24 hours at 4°C, dehydrated in a series of ethanol and xylene gradients, infiltrated with, and embedded in paraffin. *In situ* hybridization was performed with ^35^S-labeled riboprobes on 5 μm sections for normal developing brain as previously described [47] with a hybridization and wash temperature of 58°C and 63°C, respectively. Sense probes were also generated and hybridized in a similar manner to document anti-sense probe specificity (data not shown). Following autoradiography (Kodak BioMax MR film), the slides were counter-stained with Hoechst 33258 dye (2 mg/ml) to highlight nuclei and then visualized and digitally recorded (Sony DKC-5000) on a fluorescence compound microscope. We used darkfield illumination for specific expression and DAPI filtered-fluorescence for nuclear localization and general morphology. The two images were then overlaid and pseudo-colored in Adobe Photoshop 6.0.

### Northern Blot and Densitometry

Total RNA from stage HH 15, 25, 36 and 45 (E2.5, 5, 10, 20, respectively) chick brain was isolated using RNA BEE (Tel-Test) as per the manufacturer's protocol. PolyA RNA was extracted from the total RNA using Oligotex Spin Column mRNA kit (Qiagen). Two micrograms of Millenium Marker-F (Ambion) was run with the mRNA samples to indicate relative band size. The RNA samples and 1% formaldehyde gel were prepared as suggested in the Qiagen Oligotex Handbook Appendix G and transferred to a nitrocellulose membrane in alkaline conditions [48]. A ^32^P dCTP labeled probe of the 219 bp, common *NOGO *or *β-actin *fragment was prepared using the High Prime labeling kit (Roche). Hybridization of the labeled probe was performed at 68°C for 1 hour in QuickHyb Hybridization Solution (Stratagene) as per the manufacturer's protocol. The membrane was exposed to Kodak BioMax MR film at -80°C prior to developing. Controls for mRNA were performed using a PCR generated chick β-actin plasmid as described [49]. Densitometry readings of the autoradiographic film were taken using a Kodak MultiImage Light Cabinet and assessed on ChemiImager 4400 v5.1 software program using spot densitometry.

### Immunohistochemistry (IHC)

Sections adjacent to those processed for ISH were de-paraffinized and subjected to antigen retrieval (Citra Plus, BioGenex Microwave Antigen Retrieval System). Immunostaining was performed by the BioGenex i6000 (Model 1.0) automated staining system using standard immuno-protocols. Briefly, after blocking (3% H_2_O_2 _in 10% methanol and Powerblock, BioGenex), primary antibodies were applied to the slides for 40 minutes using Vimentin (1/200, Ventana), Neurofilament M (1/3200, Chemicon), GFAP (8 mg/ml, Chemicon), and PCNA (1/200, Dako). A multi-species, biotinylated, secondary antibody (Multi-link, Biogenex) was then applied to the slides for 20 minutes. The detection signal was amplified with horseradish peroxidase-conjugated streptavidin (Superlabel, BioGenex) and then visualized using a permanent (non-aqueous) chromogen, Romulin AEC (Biocare). Positive and negative (BSA) controls were performed for each staining run (Data not shown).

### Bead Implantation

Heparin acrylic beads (Sigma) of 80–120 μm size were manually isolated using a stereomicroscope, washed multiple times in PBS, soaked in 50 μg/ml FGF-4 (R&D Systems) at 4°C for 1–2 hr and then washed in PBS prior to implant. HH4 chick embryos were cultured using the EC culture method [50]. Using a 0.01 mm sharpened tungsten needle beads were pushed between endoderm and ectodermal layers of ventral side up embryos and placed in anterior, non-neural ectoderm [51]. Embryos were then harvested in MEMFA at 3, 6, or 9 hours post implant and processed for whole mount *in situ* hybridization as described above.

## Authors' contributions

SC carried out the *in situ* hybridization, northern and bead implant studies, participated in the sequence alignment and drafted the manuscript. CP participated in the northern analysis and bead implants. NM participated in the bead implants. EY and DM participated in sequence isolation and alignment. BL participated in the design of the study. KO conceived of the study, and participated in its design and coordination and helped to draft the manuscript. All authors, excluding BL, read and approved the final manuscript.

## Supplementary Material

Additional File 1 **Complementary *SHH *and *NOGO-A *gene expression in the HH6 chick embryo**. *NOGO-A *expression is prominent in the neural tissue surrounding the notochord and within the right side of the primitive node (Hensen's node). SHH is complementarily expressed having intense expression within the notochord and overlying neural tissue and also within the left side of the primitive node. Scale bar 250 μm. Click here for file

Additional File 2 ***NOGO-A *expression in CNS associated ganglia**. A) H&E and B) in situ hybridization (ISH) of *NOGO-A *expression in E5 (HH25) dorsal root ganglia (DRG). DRG is positive for *NOGO-A *expression. C) H&E and D) ISH of E10 (HH35) cranial nerve ganglia and brainstem. The ganglia is intensely positive for NOGO-A as compared to the surrounding cartilage (C) and fiber tracts (FT). Scale bar 250 μm. BS-brainstem, NC-notochord, NT-neural tube, G-ganglia Click here for file
