# Supplementary figures and images for: NOGO-A induction and localization during chick brain development indicate a role disparate from neurite outgrowth inhibition
Source: BMC Dev Biol. 2007 Apr 14;7:32. doi: 10.1186/1471-213X-7-32 (PMC1865376; doi:10.1186/1471-213X-7-32)

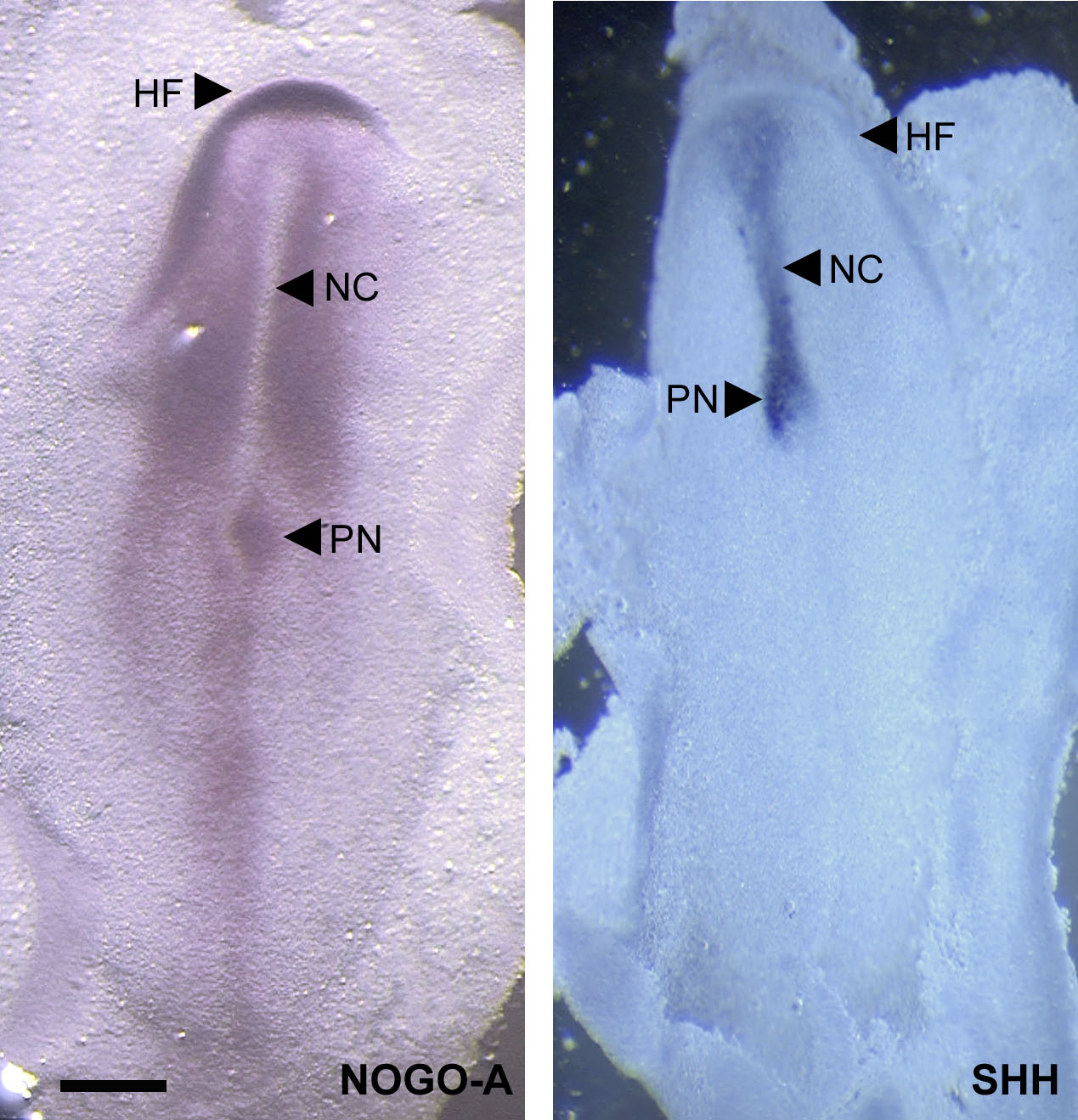

Supplement: Additional File 1 — Complementary SHH and NOGO-A gene expression in the HH6 chick embryo. NOGO-A expression is prominent in the neural tissue surrounding the notochord and within the right side of the primitive node (Hensen's node). SHH is complementarily expressed having intense expression within the notochord and overlying neural tissue and also within the left side of the primitive node. Scale bar 250 μm. [file 1471-213X-7-32-S1.png]

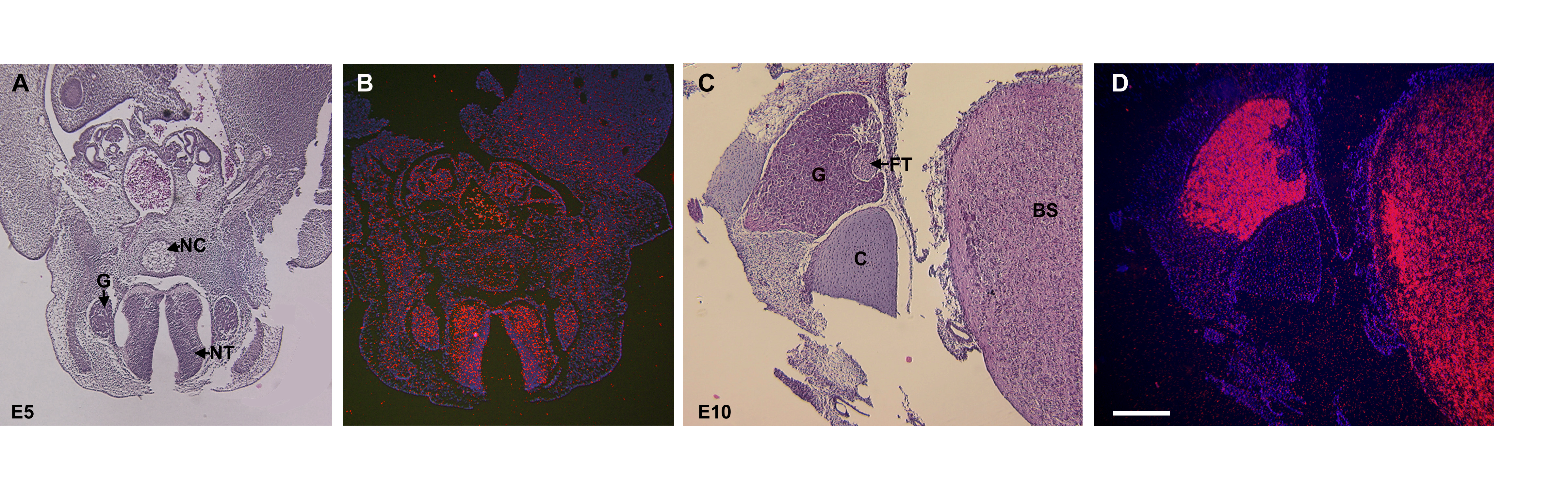

Supplement: Additional File 2 — NOGO-A expression in CNS associated ganglia. A) H&E and B) in situ hybridization (ISH) of NOGO-A expression in E5 (HH25) dorsal root ganglia (DRG). DRG is positive for NOGO-A expression. C) H&E and D) ISH of E10 (HH35) cranial nerve ganglia and brainstem. The ganglia is intensely positive for NOGO-A as compared to the surrounding cartilage (C) and fiber tracts (FT). Scale bar 250 μm. BS-brainstem, NC-notochord, NT-neural tube, G-ganglia [file 1471-213X-7-32-S2.png]
